# The Effect of Heparin-VEGF Multilayer on the Biocompatibility of Decellularized Aortic Valve with Platelet and Endothelial Progenitor Cells

**DOI:** 10.1371/journal.pone.0054622

**Published:** 2013-01-24

**Authors:** Xiaofeng Ye, Haozhe Wang, Jingxin Zhou, Haiqing Li, Jun Liu, Zhe Wang, Anqing Chen, Qiang Zhao

**Affiliations:** Department of Cardiac Surgery, Ruijin Hospital, Shanghai Jiaotong University, Shanghai, People’s Republic of China; National University of Ireland, Ireland

## Abstract

The application of polyelectrolyte multilayer films is a new, versatile approach to surface modification of decellularized tissue, which has the potential to greatly enhance the functionality of engineered tissue constructs derived from decellularized organs. In the present study, we test the hypothesis that Heparin- vascular endothelial growth factor (VEGF) multilayer film can not only act as an antithrombotic coating reagent, but also induce proliferation of endothelial progenitor cells (EPCs) on the decellularized aortic heart valve. SEM demonstrated the adhesion and geometric deformation of platelets. The quantitative assay of platelet activation was determined by measuring the production of soluble P-selectin. Binding and subsequent release of heparin and VEGF from valve leaflets were assessed qualitatively by laser confocal scanning microscopy and quantitatively by ELISA methods. Human blood derived EPCs were cultured and the adhesion and growth of EPCs on the surface modified valvular scaffolds were assessed. The results showed that Heparin-VEGF multilayer film improved decellularized valve haemocompatibility with respect to a substantial reduction of platelet adhesion. Release of VEGF from the decellularized heart valve leaflets at physiological conditions was sustained over 5 days. In vitro biological tests demonstrated that EPCs achieved better adhesion, proliferation and migration on the coatings with Heparin-VEGF multilayer film. Combined, these results indicate that Heparin-VEGF multilayer film could be used to cover the decellularized porcine aortic valve to decrease platelet adhesion while exhibiting excellent EPCs biocompatibility.

## Introduction

Heart valve disease remains a major medical problem of heart disease, and surgery offers good results for patients with advanced valvular heart disease. However, currently available heart valve prostheses have limitations, which are mainly related to life-long anticoagulation of mechanical valves and the degeneration of biological prostheses [1]. To overcome these limitations, a tissue engineered heart valve (TEHV), which is constructed by seeding cells on a valvular scaffold, might be a future option for valve replacement [2]. Decellularized allograft valves have already been shown with promising clinical results [3]. However, clinical trials using decellularized xenogenic valves were catastrophic, with severe inflammation and fibrosis of the scaffold *in vivo* [4]. More recent clinical trials showed inconsistent results. Konertz W et al. [5] supported the use of decellularized xenogenic valves as a viable alternative for right ventricular outflow tract (RVOT) reconstruction, while Perri G et al. [6] suggested that the use of xenogeneic Matrix conduits should be considered with caution.

Some studies suggested that a number of thrombogenic and inflammatory stimuli are still active within decellularized porcine aortic valve (DPAV), causing graft failure [7], [8]. Until now several approaches have been used to decrease the thrombogenicity and inflammation properties of the decellularized valve [9]–[11]. One approach has utilized polyelectrolyte multilayer films as an antiplatelet coating reagent for decellularized aortic heart valves. Our previous study indicated that the self-assembled deposition of heparin-chitosan multilayer on DPAV could be achieved with excellent hemocompatibility *in vitro* [11]. Moreover, heparin-chitosan modified DPAV can be covered by blood derived endothelial progenitor cells (EPCs). However, spontaneous endothelialization on DPAV in vivo, especially in humans, remains unresolved. Because of partial endothelialization on the valve prosthesis, patients will risk thrombus of inflammation on the valve scaffold. Therefore, hastened development of a functional endothelium will be critical question to keep the valve from deterioration.

It is well recognized that vascular endothelial growth factor (VEGF) is essential for the adhesion and growth of EPCs. Polyelectrolyte multilayer (PEM) films, built using the layer-by-layer (LbL) technique, are attractive for releasing controlled amounts of potent growth factors. In present study, we test Heparin-VEGF multilayer film as an antiplatelet coating reagent for decellularized aortic heart valve, with the potential synergistic effect of VEGF to improve the attachment and proliferation of human EPCs. To demonstrate the effects of the PEM, the adhesion, migration and growth of human blood derived EPCs on the surface modified DPAV were assessed. The modified valve scaffold was also evaluated with respect to its interaction with platelets.

## Materials and Methods

### The Decellularization Process

Fresh porcine aortic heart valve was obtained from a slaughterhouse (Shanghai Fuxin slaughterhouse, permission was obtained from this slaughterhouse to use these animal parts) and washed three times with D-Hanks solution. After being incubated for 24 h with D-Hanks containing antibiotics to limit the growth of bacteria (Cefazolin 1.0 mg/ml, Gentamicin 0.4 mg/ml, Amphotericin B 0.5 mg/ml, and Metronidazol 1.2 mg/ml), the valve was incubated with D-Hanks solution with 0.5% Triton X-100 (0694, Amresco), 0.5% sodium deoxycholate, and 0.02% EDTA (0245, Amresco) at 4°C for 48 h under continuous shaking. Then, the valve was incubated with ribonuclease A (20 mg/ml) (9001994, Sigma-Aldrich) and deoxyribonuclease (0.2 mg/ml) (DN25, Sigma-Aldrich) at 37°C for 2 h to remove cellular components [12]. The valve was thoroughly washed with D-Hanks solution and stored in liquid nitrogen until use.

### Deposition of Heparin-VEGF PEM onto Decellularized Porcine Aortic Valve

The cryopreserved valves were thawed in 37°C water and were incubated in 0.1 wt% heparin sodium salt (HEP, H4784, Sigma-Aldrich) aqueous solution for 15 min. The valves were extensively rinsed with phosphate buffered saline (PBS). The heparinized valves were then placed for 15 min into 1 µg/ml VEGF (Recombinant Human VEGF165, 100–20, PeproTech), followed by the same rinsing procedures. In accordance with this previous procedure, the HEP and VEGF were adsorbed alternatively for 15 min, with 2 consecutive adsorption steps being separated by 3 rinsing steps of 15 min with buffer. Several bilayers of HEP and VEGF were prepared by repeating the deposition process mentioned above, to produce a stable polyelectrolyte multilayer film on decellularized porcine aortic valve (PEM-DPAV). The outermost layer was VEGF. Samples with one, three and five bilayers PEM were prepared for the following experiments. Untreated decellularized porcine aortic valve (UnDPAV) also were subjected to several rinses with buffer and used as control. After PEM build-up, valves were stored at 4°C.

### Microscopic Observation

The decellularized porcine aortic valve was fixed in 10% formaldehyde, dehydrated in a graded series of ethanols and xylene, and subsequently embedded in paraffin. Serial sagittal sections of 5 µm were cut and mounted onto glass slides for histological analysis. To determine general morphology, serial sections were stained with hematoxylin and eosin (H&E) stain. For scanning electron microscopy (SEM), the specimens were fixed in 2% glutaraldehyde and then dehydrated through a graded series of ethanol concentrations (30–100%). The specimens were dried using a 50% alcohol–hexamethyldisilazane (HMDS) solution for 10 min and then in pure HMDS for 10 min, and finally air-dried in a desiccator for 12 h. The dried specimens were sputter coated with gold before examination under SEM (Hitachi S2520, Hitachi Corp., Tokyo, Japan).

### Visualization of Heparin-VEGF Multilayer

The valves were incubated with 0.1 wt% FITC-HEP (H-7482, invitrogen) and 1 µg/ml VEGF (Recombinant Human VEGF165, 100–20, PeproTech) alternately to build PEM on the surface of the valve. Then the valves were embedded in optimum cutting temperature compound (Sakura, 4583, Tokyo, Japan), flash-frozen in liquid nitrogen, and sliced into 4 µm sections using a cryostat (CM-1850; Leica) and applied to poly-L-lysine–coated slides. For visualization of VEGF binding, the sections were incubated with 1 ng/ml VEGF antibody (ab52917, Abcam, USA) in PBS solution, followed by Rhodamine -conjugated secondary antibodies (Molecular Probes, Eugene, OR, USA). Then the sections were rinsed with PBS to remove the nonadsorbed, and examined in the green and red channel of LCSM (LCSM; Leica TCS SP2, Germany). All procedures were carried out in the dark.

### Platelet Adhesion and Activation Tests

Human platelet-rich plasma (PRP) was prepared by centrifugation from citrated whole blood from healthy adult volunteer (The study was approved by the regional ethics committee, Ruijin Hospital, Shanghai Jiaotong University School of Medicine, and written consent had been obtained). Each PEM-DPAV and UnDPAV (n = 8) was incubated with 2 ml PRP (1 × 10^8^ cells/ml) for 1 h at 37°C under static conditions. The number of adherent platelets was determined by detecting the amount of lactate dehydrogenase (LDH) present after cell lysis as previously described [11]. Briefly, the suspension was aspirated and each well was rinsed carefully three times with PBS. After the PRP was removed using a vacuum aspirator, the membrane was rinsed three times with PBS and then 0.05 wt % Triton-X 100 to lyse the adherent platelets. The LDH activity in the lysed platelet suspensions was measured using an LDH cytotoxic Assay Kit (Cayman Chemical Company, Ann Arbor, MI, USA). The result of blood platelet adhesion in vitro was observed through SEM. Briefly, adherent platelets were fixed using 2.5% glutaraldehyde in PBS for at least 2 h, dehydrated in a graded series of ethanol, and freeze-dried. The samples were then sputter coated with a 7 nm layer of gold and observed using SEM. The platelet activation of the PEM-DPAV was evaluated via detection of soluble P-selectin method, as previously described [13]. PEM-DPAV and UnDPAV samples (n = 8) were incubated with 200 µl of whole blood for 1 h at 37°C under static conditions. The blood was transferred to a 1.5 ml tube and EDTA added to a final concentration of 10 mM. The sample was subsequently centrifuged at 2000 g for 10 min to obtain the plasma. The concentration of sP-selectin levels in the plasma was determined using ELISA kit (BBE 6, Human soluble P-selectin Immunoassay, R&D Systems, USA).

### Release of VEGF from PEM-DPAV

To determine the release of VEGF from Heparin-VEGF multilayer film, circular pieces of PEM-DPAV (diameter 8 mm) were incubated in 1 ml of PBS under gentle agitation. At a series of pre-determined time points (1, 2, 3, 5, 7 days, n = 3), scaffolds were transferred into new centrifuge tubes containing fresh 1 ml 1× PBS. The previous centrifuge tubes along with the 1× PBS were frozen down and stored at −20°C. Samples were analyzed using ELISA kits (R&D Systems, USA) according to manufacturer instructions. Release samples from consecutive time points were then used in cellular assays.

### Circulating Progenitor Cells Isolation, Expansion, and Characterization

The cells are from citrated whole blood from healthy adult volunteers (The study was approved by the regional ethics committee, Ruijin Hospital, Shanghai Jiaotong University School of Medicine, and written consent had been obtained). Cells were isolated from 20 ml of venous blood by density gradient centrifugation with Histopaque-1077 (10771, Sigma). Immediately after isolation, total MNC were plated on fibronectin coated cell culture flasks (1% fibronectin dissolved in PBS, F0895, Sigma) with a cell density of 1 × 10^6^ cells/cm2. The cells were maintained for 4 days in EBM-2 media (Lonza) supplemented with EGM-2-SingleQuots and 10% fetal bovine serum (FBS). Additionally, the cell culture medium was supplemented with ascorbic acid (final concentration. 75 ng/mL) and hydrocortisone (0.2 µg/mL). After 4 days of culture non-adherent cells were removed by a thorough washing with phosphate buffered saline (PBS), and the adherent cells were detached with trypsin/EDTA (T3924, Sigma). To detect the uptake of 1,1′-dioctadecyl-3,3,3′,3′-tetramethylindocarbocyanine-labeled acetylated LDL (DiI-acLDL), EPCs were incubated with DiI-acLDL (2.4 µg/mL) (L-3484, invitrogen) in EBM2 medium at 37°C for 1 h. They were then fixed with 1% paraformaldehyde for 10 min and incubated with 10 µg/ml FITC-labeled Ulex europaeus agglutinin-1 (UEA-1) (L9006, Sigma) for 1 h. Dual-staining cells positive for both DiI-acLDL and UEA-1 were judged as EPCs. An aliquot of these cells was also further characterized by FACS analysis (FACS-Calibur (Becton-Dickinson) using the following antibodies: anti-CD 31 (550389, BD Biosciences), anti-CD 45 (340040, BD Biosciences), anti-CD 133 (130-090-826 Miltenyi Biotec) and Vascular Endothelial Growth Factor-R2 (FAB357A, R&D Systems).

### EPC Adhesion and Proliferation Assay

The assay was done as described before [14]. A EPCs suspension was made by trypsinizing the plate, centrifuging at 1000 rpm for 5 min to obtain a cell pellet, and resuspending in growth media. Cell suspension densities were determined using a Multisizer 3 Coulter Counter (Beckman Coulter Inc., Fullerton, CA). For experiments using low seeding densities, 1.0 × 10^4^ cells were added per well. Seeding was performed in 96-well flat-bottom plate. Scaffolds were placed in the center of the wells (N = 6), and cell suspension was added such that the desired cell number was delivered in 200 µl of growth media. The plate was incubated at 37°C for 2 h, 24 h and 48 h to evaluate cell attachment and proliferation, respectively. The unattached cells were removed by washing with PBS three times. After washing the scaffolds with PBS three times in the initial 96-well plate, we moved the scaffolds to a new 96-well plate to avoid the influence of the cells attached to the previous wells. The number of attached cells was assayed by CCK-8 (CK04, Dojindo Laboratories, Japan) according to the manufacturers’ instructions. The optical density (OD) at 450 nm was measured with an ELISA reader. The optical density values were determined at least in triplicate. The values corresponded to the viable cell population on the scaffold in each well.

### EPCs Migration Assay

To prove the PEM-DPAV can induce migration of EPCs, transwell chamber assay was used as described before [14]. In brief, scaffolds were placed at the center bottom of the lower chamber of a 24 well Transwell (3422, Corning, Corning, NY, USA). Human EPCs were detached with trypsin; 1×10^5^ EPCs in 100 µl EGM-2 media (0.5% FBS) were placed in the upper chamber. Then, 600 µl EGM-2 media containing 0.5% FBS were placed into the lower chamber. After 6 h incubation at 37°C, EPCs on the top of the membrane was wiped off with a cotton swab. The transwell membrane was rinsed with PBS (37°C), and then incubated in 1 µg/mL Hoechst 33342 (Invitrogen, CA, USA) at room temperature for 10 min. Migration of EPCs was evaluated by measuring the migrated cells in six random high-power (100×) microscope fields, and the average of these six fields were taken. Experiments were repeated six times.

### Measurement of Nitric Oxide (NO) Production from Seeded EPCs

Same number of EPCs were seeded on UnDPAV or PEM-DPAV and cultured as described above. The complete EGM-2 medium was replaced with phenol red-free complete M-199 (as recommended by the manufacturer of the Nitrite Assay Kit) for this study. After 24 hours, the cell culture medium was collected, frozen at −80°C, and freeze-dried. The lyophilized medium was dissolved in 5 µl DI water, and the nitrite concentrations in these samples were measured using Measure-iTTM High-Sensitiviy Nitrite Assay Kit following the manufacturer’s instructions.

### Immunofluorescence Observation of VEGFR2 Clustering

Endothelial cells on PEM-DPAV were fixed with 4% paraformaldehyde for 15 min at room temperature in the absence of permeabilization. Antibody against the extracellular domain of VEGFR2 was diluted 1∶100 in 1% BSA/PBS. Tissues were then examined under a confocal microscope (LCSM; Leica TCS SP2, Germany) under a 60× oil objective.

### Statistical Analysis

All data were presented as mean ± SEM. Statistical analysis for determination of differences in the measured properties between groups was accomplished using a one-tailed analysis of variance and Student’s t-test, performed with a computer statistical program (SPSS 18.0 for Windows; SPSS, Chicago, IL), and p values<0.05 were considered statistically significant.

## Results

### Morphology of DPAV and PEM-DPAV

**Figure 1:**
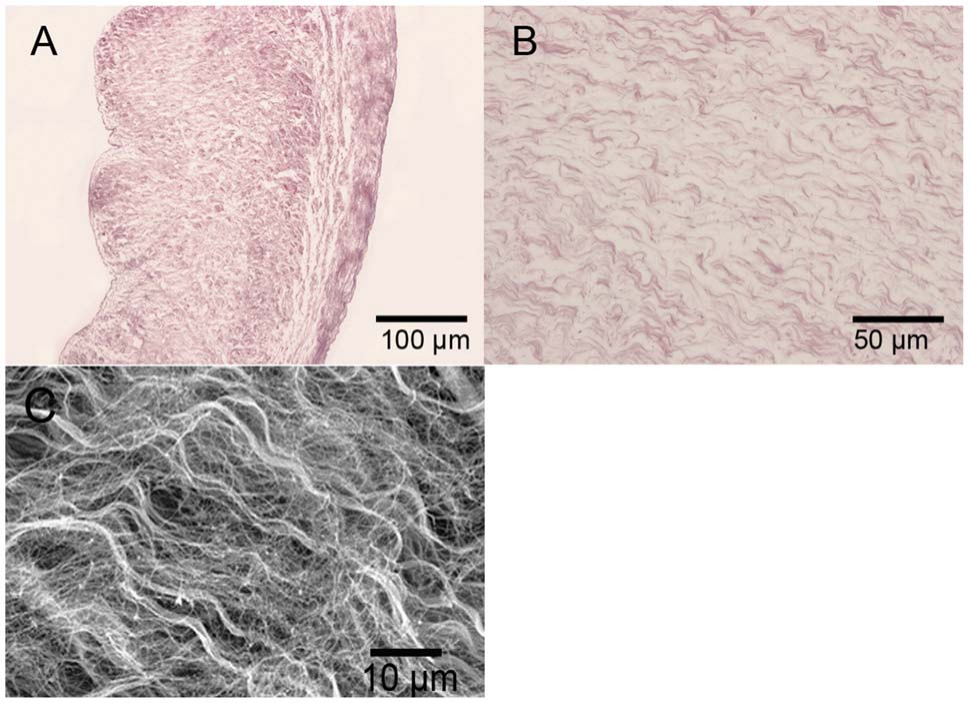
Histology of DPAV were analyzed with H&E and SEM. H&E photomicrographs (A, B) and SEM (C) of decellularized porcine aortic valve, all showing that the cellular components of the porcine aortic heart valve leaflets were completely removed. The wave-like collagen fiber was conserved. (A) Scale bars: 100 µm; (B) scale bar: 50 µm; (C) scale bar: 10 µm.

**Figure 2:**
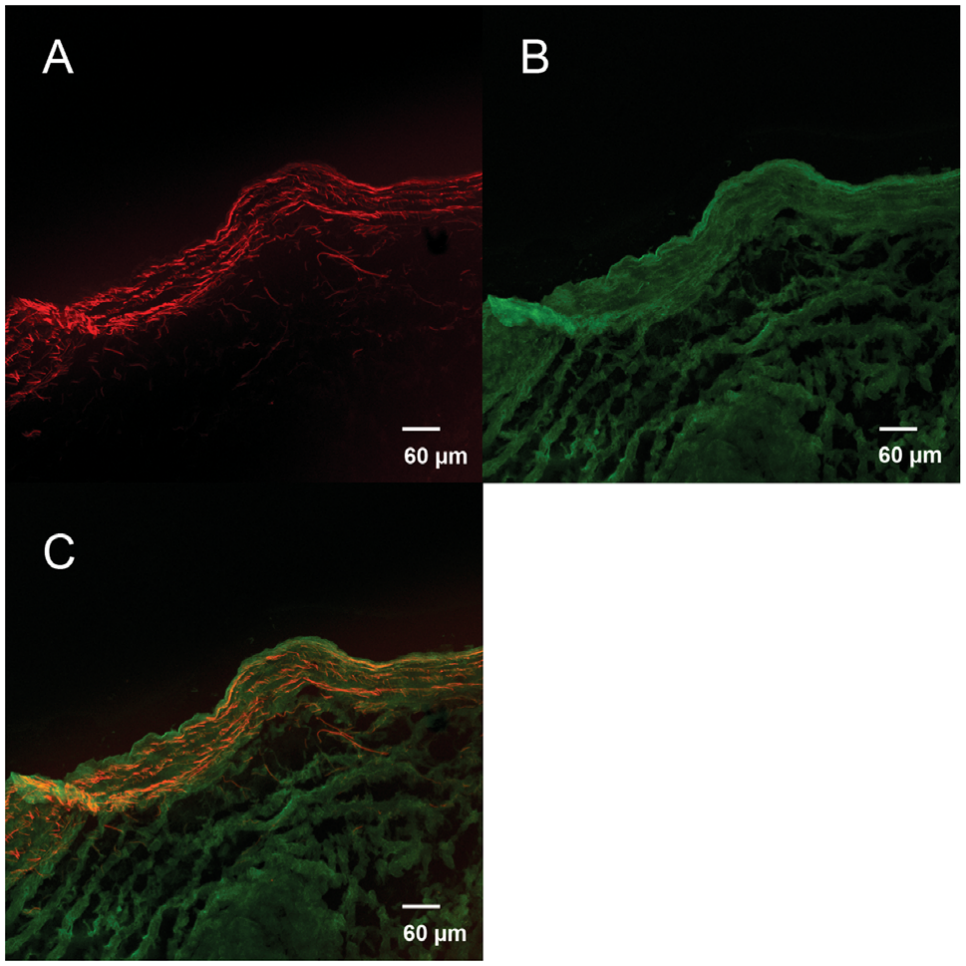
The fluorescence mainly distributed on the surface of the PDAV. The red fluorescence indicated the presence of VEGF bound to HEP-PDAV (A), while the green fluorescence distributed on the surface of the tissue cross section represent HEP bound to the whole the decellularized valve (B). The fluorescence between FITC and Rhodamine B was non-overlapping, indicating that the both HEP and VEGF were mutually bound to the PDAV (C). (A–C) Scale bars: 60 µm.

Decellularization completely removed the cellular components of the porcine aortic heart valve leaflets (Fig. 1A, 1B). SEM also found no cell remnant among the wave-like collagen fibers, confirming the success of decellularization (Fig. 1C). The red fluorescence distributed on the surface of the tissue cross section indicate the presence of VEGF bound to the decellularized valve (Fig. 2A). The positive green fluorescence of HEP-FITC indicated that HEP was bound to the HEP-DPAV (Fig. 2B). Unlike the red fluorescence, HEP bound the entire valve scaffold. And the combination is showed in Fig. 2C.

### Platelet Adhesion and Activation

**Figure 3:**
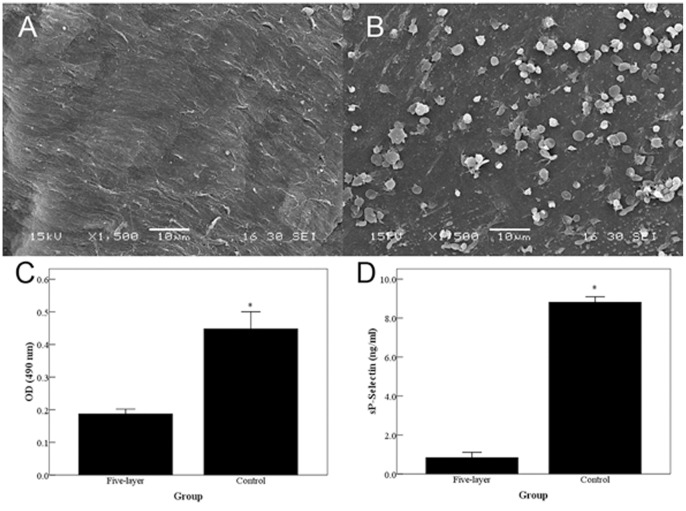
SEM images of human platelet-rich plasma (PRP) incubated with DPAV. There are many adhered platelets on UnDPAV (B) as compared to five-layer groups (A). Platelets on UnDPAV demonstrated aggregate and changed morphology. The number of adhered platelets on valves as was determined by quantification of LDH activity (C). Platelet activation is measured by sP-selectin levels (D). * Corresponds to a p<0.001 of five-layer PEM-DPAV in comparison to UnDPAV. (A, B) scale bars: 10 µm.

Since the xenogenous valvular ECM may remain thrombogenic, the comparison of platelet adhesion and activation were performed between PEM-DPAV and UnDPAV. Apparently, few platelets adhered to five-layer PEM-DPAV (Fig. 3A), comparing with UnDPAV (Fig. 3B). Platelets that adhered to UnDPAV demonstrated aggregate and changed morphology. The number of platelets that adhered to DPAV was also quantified by measuring LDH activity. Compared with UnDPAV, five-layer PEM-DPAV significantly reduced the number of adhered platelet (Fig. 3C, p<0.001). Measurements of sP-selectin were performed to determine the level of platelet activation. Significantly more sP-selectin was detected from samples incubated with UnDPAV, as compared to five-layer PEM-DPAV (Fig. 3D, p<0.001).

### Controlled Release of VEGF from PEM-DPAV

**Figure 4:**
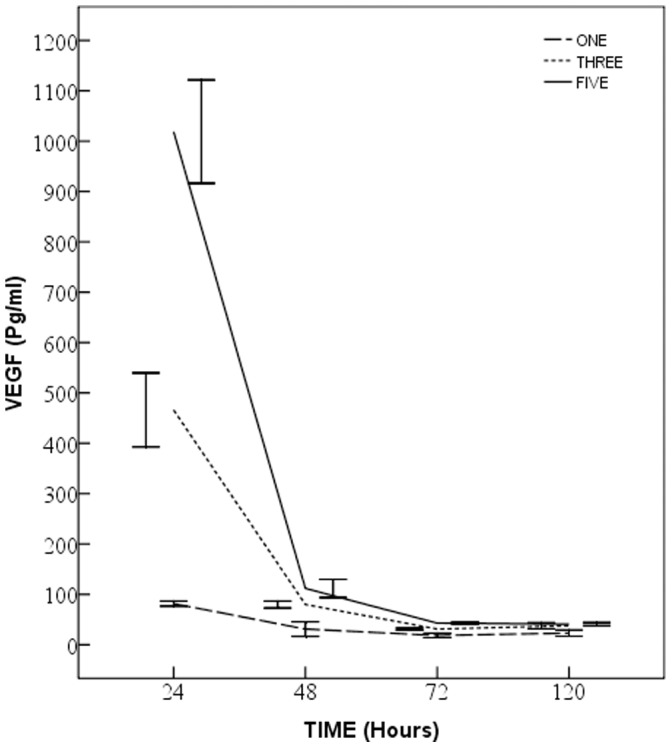
In vitro release of VEGF from valvular scaffolds coated with one, three and five layers of Heparin-VEGF. The amounts of the released VEGF differ significantly in the first 24 h (p<0.001). In the next 48 h, they still had difference (p<0.05), but the difference reduced. At 120 h, the three and five-layer groups still release much more VEGF than the one-layer group (p<0.05), while the difference between the two groups is no longer significant (p>0.05).

The in vitro release of VEGF from modified valve is shown in Figure 4. The short-term kinetic study showed different initial burst releases among one-layer, three-layer and five-layer groups within first 120 h of incubation. The amount of the released VEGF decreased significantly (p<0.001) and then remained stable after 72 h (p>0.05). In the first 24 h, the five-layer group released about 10 times higher amount of VEGF as compared to one-layer group (p<0.001). The significant difference among groups remained at 72 h (p<0.05). At 120 h the three and five-layer groups still release much more VEGF than the one-layer group (p<0.05), though the difference between the two groups is no longer significant (p>0.05).

### EPC Adhesion, Proliferation and Migration

**Figure 5:**
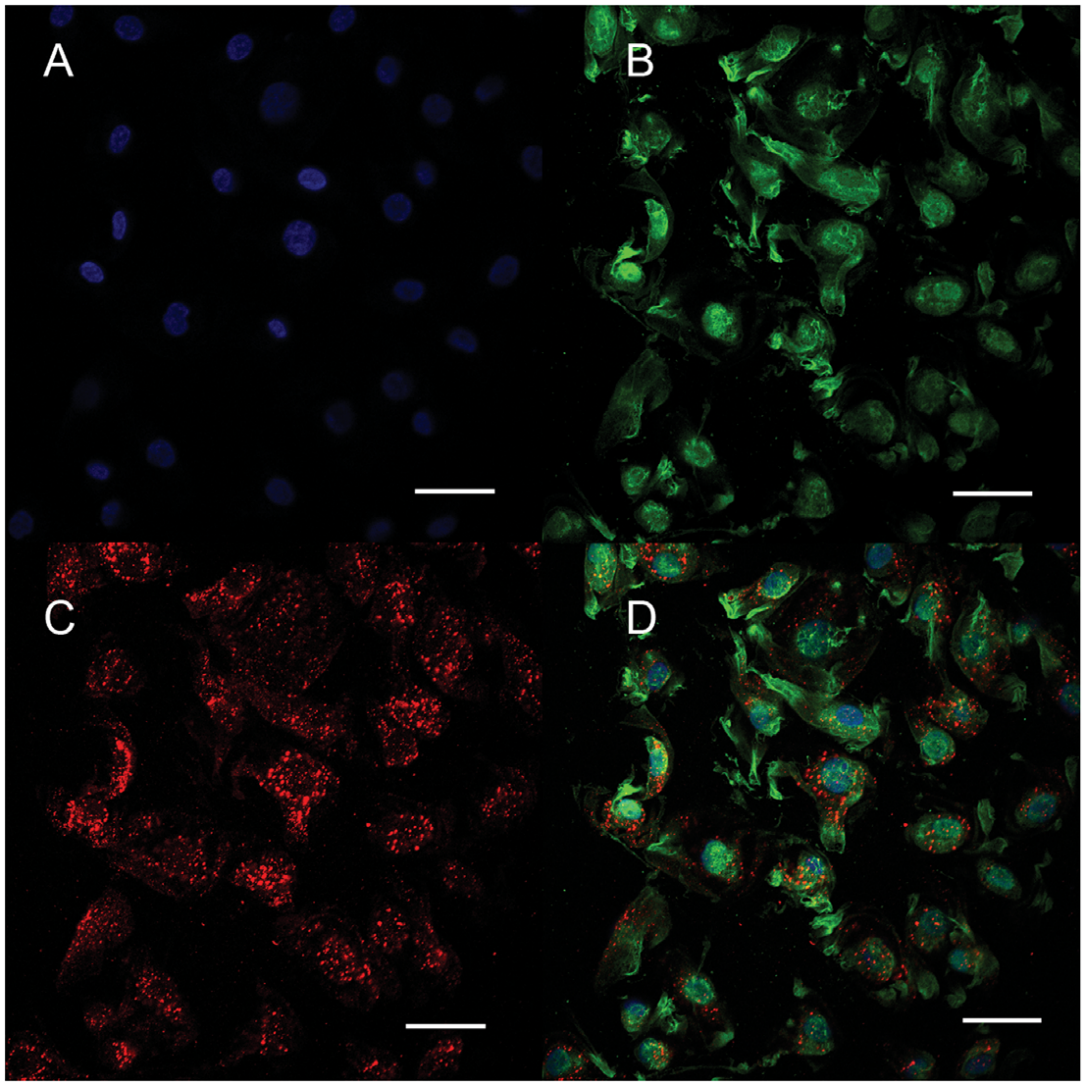
Immunofluorescence staining of EPCs. The cellular nuclear was stained with DAPI (A, blue). The cells were capable of UEA-1 lectin binding (B, green) and cellular uptake of acLDL (C, red), both of which are features of endothelial lineage (D, merged). (A–D) scale bars: 50 µm.

**Figure 6:**
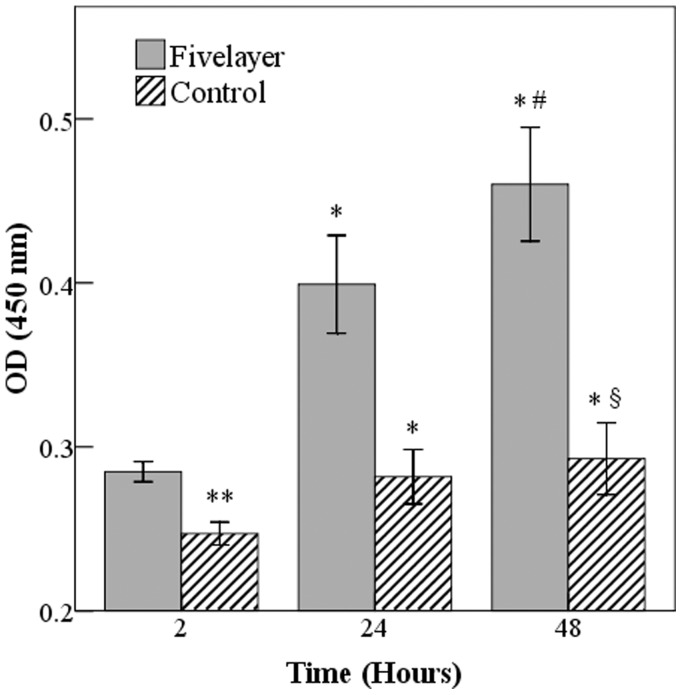
The attachment and proliferation of EPCs on the PEM-DPAV and UnDPAV. The number of cells on PEM-DPAV, which is represented by OD value, was much more at each time points (p<0.001), * Corresponds to a p<0.001 of the cell number at 24 h and 48 h in comparison to the cell number at 2 h in the each group; ** Corresponds to a p<0.05 of PEM-DPAV in comparison to UnDPAV at 2 h; whereas # indicates a p<0.001 of the cell number at 48 h in comparison to the cell number at 24 h in PEM-DPAV group; and § indicates a p<0.05 of the cell number at 48 h in comparison to the cell number at 24 h in UnDPAV group. Values represent the mean and standard deviation from 6 samples.

**Figure 7:**
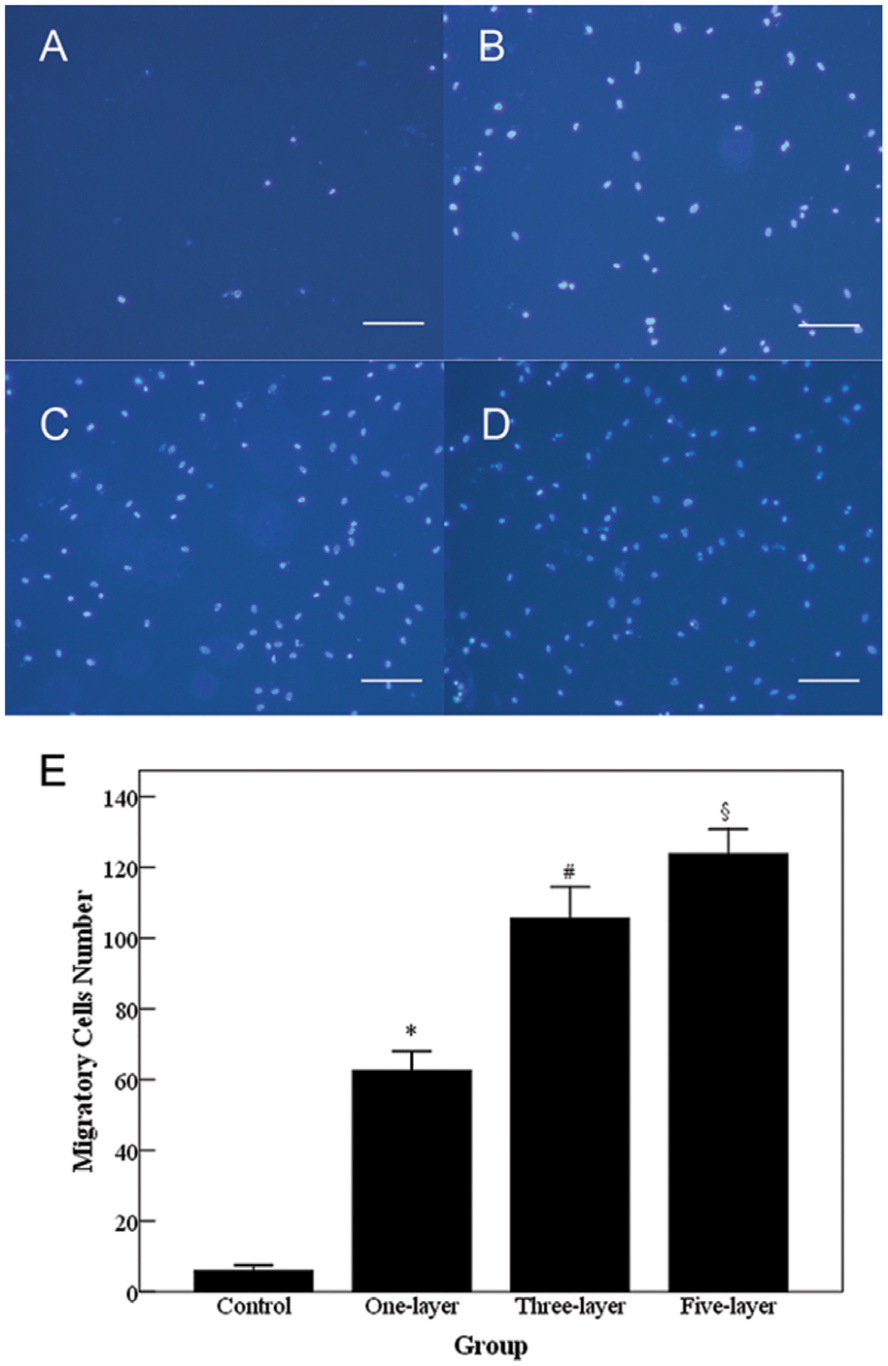
Cell migration is measured with Transwell chamber using Hoechst33342, a non-toxic specific vital stain for DNA. Scale bar 100 µm. Valve coated with heparin-VEGF multilayer increased human EPCs migration to the lower surface of the membrane of Transwell chambers. * Corresponds to a p<0.001 between UnDPAV (A) and one-layer PEM-DPAV (B); whereas # indicates a p<0.05 between one- and three-layer PEM-DPAV (C); and § indicates a p<0.05 between three- and five-layer PEM-DPAV (D). Values represent the mean and standard deviation from 6 samples.

Human EPC colonies appeared 5–7 days after isolation. Isolated EPCs exhibited characteristic cobblestone endothelial cell morphology by light microscopy. Flow cytometry confirmed presence of CD31, Vascular Endothelial Growth Factor-R2, and absence of CD45 and CD133 (data not shown). The cells were found to be double-positive for uptake of DiI-acLDL and binding of FITC-UEA-1, both of which are features of endothelial lineage (Fig. 5). To investigate the adhesion and proliferation of EPCs on the modified valve scaffold, EPCs were seeded and cultured on the valve for 48 h. EPCs exposed to PEM-DPAV exhibited an increase in the number of adhesive cells at 2 h (Fig. 6, p<0.05). The number of adhered cells began to increase in both groups in the following 48 h. However, the number of cells proliferated on PEM-DPAV was much more at each time points (Fig. 6, p<0.001). Moreover, the control group had no significant increase in cell number after 24 h (p>0.05). It is well known that VEGF can induce endothelial cell migration. We further investigated whether PEM-DPAV can affect the migrative activity of EPCs. The results demonstrated that valve coated with heparin-VEGF multilayer can increase human EPC migration, which is also increased with the number of multilayer (Fig. 7, p<0.05).

### Functional Change of EPCs on PEM-DPAV

**Figure 8:**
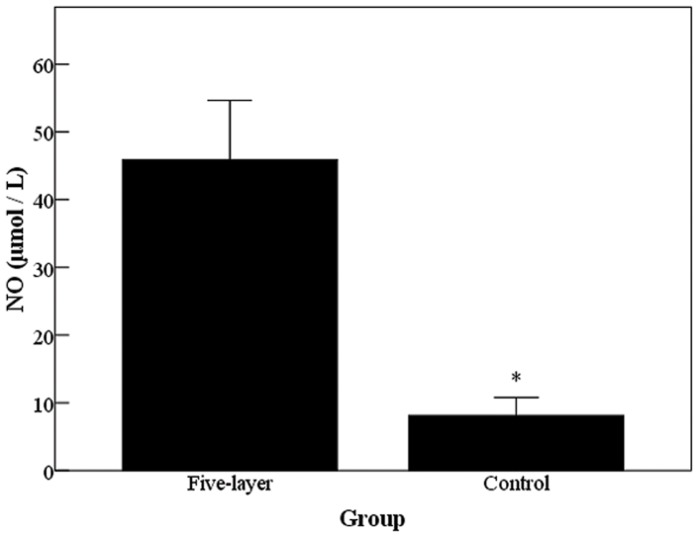
The EPCs seeded on PEM-DPAV had statistically significant more production of NO as compared with the cells on UnDPAV. * Corresponds to a p<0.001.

**Figure 9:**
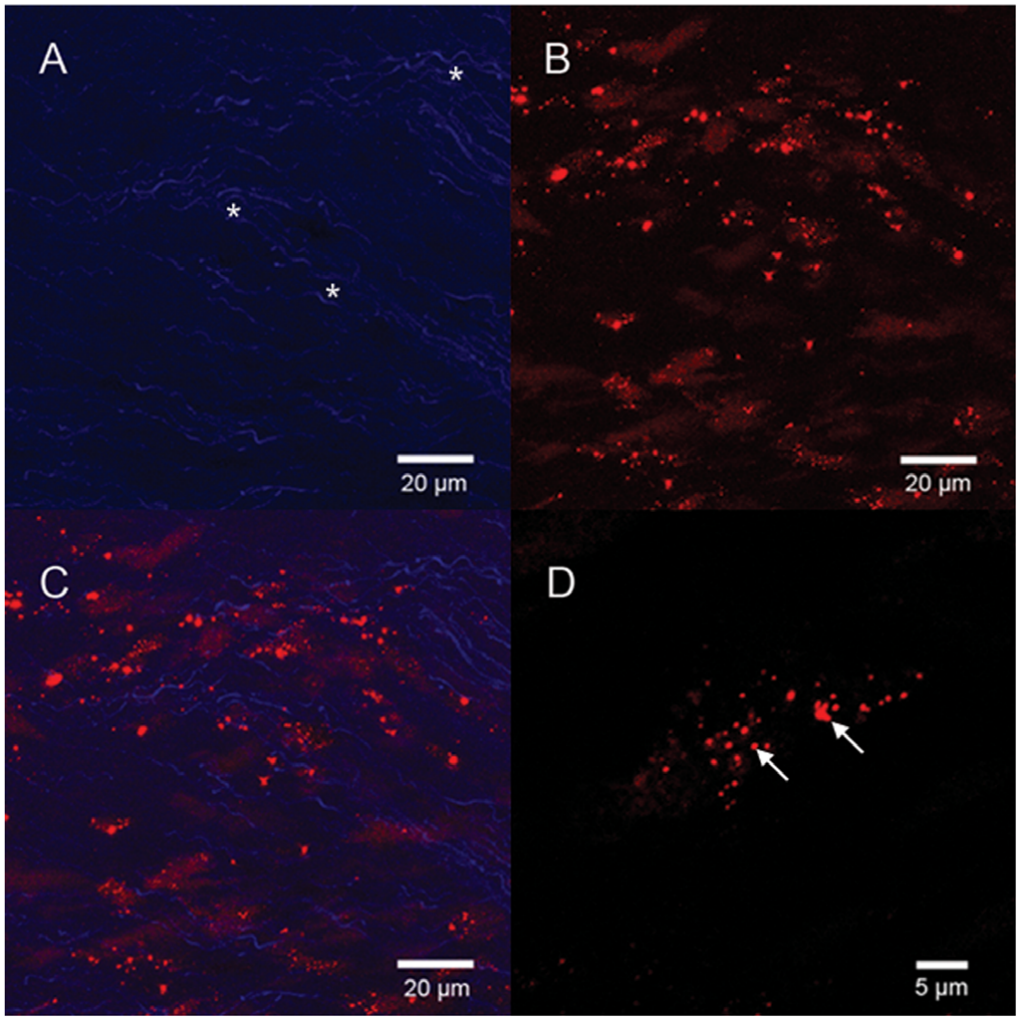
EPCs were fixed and stained using antibody against extracellular domain of VEGFR2 in the absence of permeabilization. The wave-like collagen of DPAV has autofluorescence, indicated by asterisk. The PEM-DPAV result VEGFR2 clustering (B and D, arrow indicate the clustering), and (C, merged). (A-C) Scale bars: 20 µm; (D) scale bar: 5 µm.

NO production is one of the important functions of normal valvular endothelial cells. NO produced by cells is released into the cell culture medium and quickly oxidized to nitrite. We measured the nitrite concentration in the cell culture medium as a representative of NO produced by the cells. The result showed that cells on PEM-DPAV had statistically significant more production of NO as compared with the cells on DPAV (Fig. 8, p<0.001). Therefore, it can be concluded that cells maintained their normal functions such as NO production despite the significantly higher adhesion and proliferation of EPCs grown on the PEM-DPAV. We also found that the PEM-DPAV result VEGFR2 clustering, which were less predominant in cells treated with soluble VEGF (Fig. 9).

## Discussion

Tissue engineered heart valves represent a new source of valve prosthesis, which could be viable, nonimmunogenic, and biologically active. Direct implantation of a decellularized valve graft is more clinically practical, since it circumvents the complicated procedures needed for *in vitro* culture. Because of the limited availability of homografts, decellularized xenografts have been used as an alternative valve substitute in recent years [15]. However, it was reported that the decellularized porcine valves were immunogenic and can induce inflammatory responses [6]. Surface modification provides a new way to protect the matrix from the platelet adhesion, and may also expedite the process of endothelialization *in vivo*. The aim of the present study was utilize the HEP-VEGF multilayer film to modify the surface of the decellularized valve scaffold. Results revealed that HEP-VEGF multilayer film could reduce the adhesion and activation of platelets on the scaffold. In addition, seeded EPCs demonstrated better adhesion, proliferation and migration on the treated scaffolds.

For the past two decade, PEM has been suggested as a versatile technique of surface modification [16]. Multilayer films are constructed by the alternate adsorption of oppositely charged polyelectrolytes at the surface of a material. To produce functional PEM, researchers have incorporated different bioactive factors in PEM, such as growth factors [17], DNA complexes [18] or drugs [19]. The sustained and control-released growth factor from such modified PEMs may be an alternative strategy to improve *in situ* stem cell adhesion, differentiation and growth. It was recently reported that bFGF could be loaded via layer-by-layer electrostatic deposition [20]. In present study, we used VEGF instead of bFGF because of its specificity to EPC [21]. It was observed that VEGF could be bound to DPAV, and the amount of released VEGF was directly proportional to the layer number of heparin and VEGF. Correspondingly, five-layer PEM-DPAVs were used in the following platelet and EPC experiments.

Heparin (HEP) is a glycosaminoglycan (GAG) that is covalently attached to core proteins to form proteoglycans. It can bind to proteins due to the attraction of positively charged amino acid residues in proteins to its negatively charged sulfate groups. As decellularization was accompanied by a reduction in GAG content, which function as a reservoir for growth factors [22], we reasoned that it might be beneficial to use heparin as a component of PEM, aiming to restore the growth factor in the decellularized tissue. HEP is also the most commonly used anticoagulant reagent. It has been previously observed that PEM with HEP functions in part as an anti-coagulant and drastically reduced platelet adhesion [23]–[24], and the present study is consistent with these previous findings. P-selectin is a surface-component of platelet granules that appears when platelets are activated, and in line with the results of platelet adhesion, P-selectin was significantly reduced in five-layer PEM-DPAV.

High failure rates of tissue valves arise due to the lack of endothelial cells, especially among children. One approach to this problem is the *in vitro* endothelialization of glutaraldehyde-fixed heart valves, which were demonstrated to show sustained antithrombotic properties [25]. However, several issues, including time-consuming cell-culture, limit *in vitro* repopulation with these cells. Due to the importance of endotheliazation and the difficulty in attaining it, *in vivo* endothelialization of valve prosthesis has become one of the most important current challenges for TEHV. In the present study, we successfully improved the adhesion of EPCs on the HEP-VEGF multilayer modified valve scaffold. Compared with traditional PEM films [26], our functional PEM films not only improved EPCs attachment, but also their proliferation and migration. Localized delivery of VEGF is favorable, as it keeps a high concentration around the valve and remains relatively stable in this areahttp://en.wikipedia.org/wiki/Controlled_internal_drug_release - cite_note-wheaton-3.

For thinner PEM films, biological responses have been shown to be dependent upon the terminal PEM layer [27]. The present study also addressed one of many functions of the blood-derived EPCs, which contacted with the VEGF layer of the PEM. The NO assay of the culture medium indicates that EPCs maintained the relaxation of vascular tissue, which is one of the functions of endothelial cells, The activation of VEGFR2 was distinct from that of soluble VEGF in terms of recruitment of receptor partners, which further confirmed that EPCs have been influenced by the PEM film. This result is consistent with what has previously been found on human aortic and vein endothelial cell cultured with matrix-bounded VEGF [28]. However, these preliminary data need further experiments to demonstrate the functional endothelial cell layer on the DPAV. Moreover, whether the ECM of DPAV also contributes in the in-situ EPCs’ differentiation remain to be studied.

The present study did not further explore some the biochemical and biophysical properties of the PEM film, such as topographies of the heart valve, which may affect the cells’ adhesion as well as the orientation of the seeded EPCs. Also, the influence of the film on the bioactivity of the attached endothelial cells *in vivo* was not addressed. To better understand the mechanism by which HEP-VEGF multilayer films affect the attached EPCs, substantial work is still needed, such as PEM stability, other functional characterization and dynamics, and EPCs differentiation *in vivo*.

### Conclusion

Our study demonstrates that the self-assembled deposition of HEP and VEGF multilayers on DPAV can be achieved with antiplatelet prosperity in vitro. It also demonstrated that the scaffold can be covered by blood derived EPCs. Moreover, such a modification can improve EPCs attachment, proliferation and migration. Combined, the results suggest that HEP-VEGF DPAV is a biomaterial warranting further investigation for use in TEHV.

## References

[1] Ruel M, Chan V, Bedard P, Kulik A, Ressler L, et al (2007) Very long-term survival implications of heart valve replacement with tissue versus mechanical prostheses in adults <60 years of age. Circulation 116: I294–300. 17846320 10.1161/CIRCULATIONAHA.106.681429

[2] Rippel RA, Ghanbari H, Seifalian AM (2012) Tissue-Engineered Heart Valve: Future of Cardiac Surgery. World J Surg. 36: 1581–1591. 10.1007/s00268-012-1535-y 22395345

[3] Cebotari S, Tudorache I, Ciubotaru A, Boethig D, Sarikouch S, et al (2011) Use of fresh decellularized allografts for pulmonary valve replacement may reduce the reoperation rate in children and young adults: early report. Circulation 124: S115–123. 21911800 10.1161/CIRCULATIONAHA.110.012161

[4] Simon P, Kasimir MT, Seebacher G, Weigel G, Ullrich R, et al.. (2003) Early failure of the tissue engineered porcine heart valve SYNERGRAFT in pediatric patients. Eur J Cardiothorac Surg 23: 1002–1006; discussion 1006. 10.1016/s1010-7940(03)00094-0 12829079

[5] Konertz W, Angeli E, Tarusinov G, Christ T, Kroll J, et al (2011) Right ventricular outflow tract reconstruction with decellularized porcine xenografts in patients with congenital heart disease. J Heart Valve Dis 20(3): 341–7. 21714427

[6] Perri G, Polito A, Esposito C, Albanese SB, Francalanci P, et al (2012) Early and late failure of tissue-engineered pulmonary valve conduits used for right ventricular outflow tract reconstruction in patients with congenital heart disease. Eur J Cardiothorac Surg 41(6): 1320–5. 22219487 10.1093/ejcts/ezr221

[7] Zhou J, Fritze O, Schleicher M, Wendel HP, Schenke-Layland K, et al (2010) Impact of heart valve decellularization on 3-D ultrastructure, immunogenicity and thrombogenicity. Biomaterials 31: 2549–2554. 20061016 10.1016/j.biomaterials.2009.11.088

[8] Kasimir MT, Rieder E, Seebacher G, Nigisch A, Dekan B, et al.. (2006) Decellularization does not eliminate thrombogenicity and inflammatory stimulation in tissue-engineered porcine heart valves. J Heart Valve Dis 15: 278–286; discussion 286. 16607912

[9] Stamm C, Khosravi A, Grabow N, Schmohl K, Treckmann N, et al.. (2004) Biomatrix/polymer composite material for heart valve tissue engineering. Ann Thorac Surg 78: 2084–2092; discussion 2092–2083. 10.1016/j.athoracsur.2004.03.106 15561041

[10] Wilcox HE, Korossis SA, Booth C, Watterson KG, Kearney JN, et al.. (2005) Biocompatibility and recellularization potential of an acellular porcine heart valve matrix. J Heart Valve Dis 14: 228–236; discussion 236–227. 15792184

[11] Ye X, Hu X, Wang H, Liu J, Zhao Q (2012) Polyelectrolyte multilayer film on decellularized porcine aortic valve can reduce the adhesion of blood cells without affecting the growth of human circulating progenitor cells. Acta Biomater 8: 1057–1067. 22122977 10.1016/j.actbio.2011.11.011

[12] Ye X, Zhao Q, Sun X, Li H (2009) Enhancement of mesenchymal stem cell attachment to decellularized porcine aortic valve scaffold by in vitro coating with antibody against CD90: a preliminary study on antibody-modified tissue-engineered heart valve. Tissue Eng Part A 15: 1–11. 18759669 10.1089/ten.tea.2008.0001

[13] Motlagh D, Yang J, Lui KY, Webb AR, Ameer GA (2006) Hemocompatibility evaluation of poly(glycerol-sebacate) in vitro for vascular tissue engineering. Biomaterials 27: 4315–4324. 16675010 10.1016/j.biomaterials.2006.04.010

[14] Tang Y, Huang B, Sun L, Peng X, Chen X, et al (2011) Ginkgolide B promotes proliferation and functional activities of bone marrow-derived endothelial progenitor cells: involvement of Akt/eNOS and MAPK/p38 signaling pathways. Eur Cell Mater 21: 459–469. 21623570 10.22203/ecm.v021a34

[15] Konertz W, Angeli E, Tarusinov G, Christ T, Kroll J, et al (2011) Right ventricular outflow tract reconstruction with decellularized porcine xenografts in patients with congenital heart disease. J Heart Valve Dis 20: 341–347. 21714427

[16] Boudou T, Crouzier T, Ren K, Blin G, Picart C (2010) Multiple functionalities of polyelectrolyte multilayer films: new biomedical applications. Adv Mater 22: 441–467. 20217734 10.1002/adma.200901327

[17] Shah NJ, Macdonald ML, Beben YM, Padera RF, Samuel RE, et al (2011) Tunable dual growth factor delivery from polyelectrolyte multilayer films. Biomaterials 32: 6183–6193. 21645919 10.1016/j.biomaterials.2011.04.036 PMC3202614

[18] Richard D, Nguyen I, Affolter C, Meyer F, Schaaf P, et al (2010) Polyelectrolyte multilayer-mediated gene delivery for semaphorin signaling pathway control. Small 6: 2405–2411. 20878791 10.1002/smll.201000228

[19] Berg MC, Zhai L, Cohen RE, Rubner MF (2006) Controlled drug release from porous polyelectrolyte multilayers. Biomacromolecules 7: 357–364. 16398536 10.1021/bm050174e

[20] De Cock LJ, De Koker S, De Vos F, Vervaet C, Remon JP, et al (2010) Layer-by-layer incorporation of growth factors in decellularized aortic heart valve leaflets. Biomacromolecules 11: 1002–1008. 20155947 10.1021/bm9014649

[21] Asahara T, Takahashi T, Masuda H, Kalka C, Chen D, et al (1999) VEGF contributes to postnatal neovascularization by mobilizing bone marrow-derived endothelial progenitor cells. EMBO J 18: 3964–3972. 10406801 10.1093/emboj/18.14.3964 PMC1171472

[22] Converse GL, Armstrong M, Quinn RW, Buse EE, Cromwell ML, et al (2012) Effects of cryopreservation, decellularization and novel extracellular matrix conditioning on the quasi-static and time-dependent properties of the pulmonary valve leaflet. Acta Biomater 8: 2722–2729. 22484150 10.1016/j.actbio.2012.03.047

[23] Tan Q, Ji J, Zhao F, Fan DZ, Sun FY, et al (2005) Fabrication of thromboresistant multilayer thin film on plasma treated poly (vinyl chloride) surface. J Mater Sci Mater Med 16: 687–692. 15965602 10.1007/s10856-005-2541-5

[24] Chen J, Chen C, Chen Z, Li Q, Huang N (2010) Collagen/heparin coating on titanium surface improves the biocompatibility of titanium applied as a blood-contacting biomaterial. J Biomed Mater Res A 95: 341–349. 20623672 10.1002/jbm.a.32847

[25] Schopka S, Schmid FX, Hirt S, Birnbaum DE, Schmid C, et al (2009) Recellularization of biological heart valves with human vascular cells: in vitro hemocompatibility assessment. J Biomed Mater Res B Appl Biomater 88: 130–138. 18615474 10.1002/jbm.b.31159

[26] Boura C, Menu P, Payan E, Picart C, Voegel JC, et al (2003) Endothelial cells grown on thin polyelectrolyte mutlilayered films: an evaluation of a new versatile surface modification. Biomaterials 24: 3521–3530. 12809781 10.1016/s0142-9612(03)00214-x

[27] Serizawa T, Yamaguchi M, Akashi M (2002) Alternating bioactivity of polymeric layer-by-layer assemblies: anticoagulation vs procoagulation of human blood. Biomacromolecules 3: 724–731. 12099816 10.1021/bm0200027

[28] Chen TT, Luque A, Lee S, Anderson SM, Segura T, et al (2010) Anchorage of VEGF to the extracellular matrix conveys differential signaling responses to endothelial cells. J Cell Biol 188: 595–609. 20176926 10.1083/jcb.200906044 PMC2828913

